# The role of microbiota in tumorigenesis, progression and treatment of bladder cancer

**DOI:** 10.20517/mrr.2023.47

**Published:** 2023-11-20

**Authors:** Zijing Peng, Jingming Zhuang, Bing Shen

**Affiliations:** Department of Urology, Shanghai General Hospital, Shanghai Jiao Tong University School of Medicine, Shanghai 200080, China.; ^#^Authors contributed equally.

**Keywords:** Microbiota, bladder cancer, mechanism, tumor microenvironment, microbial metabolites

## Abstract

For decades, the urinary system was regarded as a sterile environment due to the absence of any bacterial growth in clinical standard urine cultures from healthy individuals. However, a diverse array of microbes colonizes the urinary system in small quantities, exhibiting a variable compositional signature influenced by differences in sex, age, and pathological state. Increasing pieces of evidence suggest microbiota exists in tumor tissue and plays a crucial role in tumor microenvironment based on research in multiple cancer models. Current studies about microbiota and bladder cancer have preliminarily characterized the bladder cancer-related microbiota, but how the microbiota influences the biological behavior of bladder cancer remains unclarified. This review summarizes the characteristics of microbiota in bladder cancer, aims to propose possible mechanisms that microbiota acts in tumorigenesis and progression of bladder cancer based on advances in gut microbiota, and discusses the potential clinical application of microbiota in bladder cancer.

## INTRODUCTION

Bladder cancer is one of the most common malignant tumors in the urinary system, with an estimated 570,000 new cases and 210,000 deaths globally in 2020^[1]^. Risk factors confirmed by different studies include tobacco exposure, chemicals, infections, chronic inflammation, iatrogenic causes, *etc*.^[2,3]^.

In the last decade, advancements in urine culture protocols, like the enhanced quantitative urine culture (EQUC)^[4]^, and 16S rRNA gene sequencing technology, have revealed the presence of microbiota in urine, challenging the long-held belief that urine is sterile^[5,6]^. These findings suggest that the urinary system may have a microbial ecosystem similar to the gut microbiota. It is acknowledged that microbiota plays a vital role in cancer and powerful molecular evidence suggests microbiota exists within tumor tissue across at least 33 types of tumors, including bladder urothelial carcinoma^[7]^. The microbiota inside tumor tissue is demonstrated to be tumor type-specific and may directly modulate tumorigenesis, progression, and response to cancer treatment by various mechanisms^[8-12]^. Based on observational studies, alterations in the urinary microbiota have been linked to neurogenic bladder dysfunction, urge urinary incontinence, bladder cancer, and some other urinary system diseases^[6,13]^, but how the microbiota correlates with the initiation and progression of bladder cancer remains unclear.

Here, we summarize research about bladder cancer-associated microbiota, and put forward the possible underlying mechanisms that may elucidate the interaction between microbiota and bladder cancer. At last, we hypothesize the potential clinical applications of microbiota in the diagnosis, prognosis, and treatment of bladder cancer.

## MICROBIOTA AND TUMORIGENESIS OF BLADDER CANCER

### The signature of urinary and tissue microbiota in patients with bladder cancer

To investigate the relevance of microbiota in the tumorigenesis of bladder cancer, a comparison of microbiota composition between healthy individuals and bladder cancer patients is a reasonable approach [Table 1].

**Table 1:** Urinary or intratumor microbiota in BC patients and healthy individuals

**Research**	**Sample**	**Cancer group**	**Ref.**
Xu *et al*. 2014	Urine samples from 8 UC and 6 HC	Streptococcus ↑	[15]
Bučević Popović *et al*. 2018	Urine samples from 12 BC and 11 HC	Fusobacterium ↑ Actinobaculum ↑	[16]
Wu *et al*. 2018	Urine samples from 29 BC and 18 HC	Acinetobacter ↑ Anaerococcus ↑ Rubrobacter ↑ Sphingobacterium ↑	[17]
Pederzoli *et al.* 2020	29 BC tissue samples and 29 paired controls	Burkholderia ↑	[18]
Mansour *et al*. 2020	10 BC urine samples and 14 BC tissue samples	Bacteroides ↑ Akkermansia ↑ Klebsiella ↑ Clostridium sensu stricto ↑ Enterobacter ↑	[19]
Liu *et al*. 2019	22 BC tissue samples and 12 adjacent controls	Actinobacteria ↑ Proteobacteria ↑ Firmicutes ↓ Bacteroidetes ↓ Brucellaceae ↑ Acinetobacter ↑A noxybacillus↑	[21]
Parra-Grande *et al*. 2022	32 BC tissue samples and 26 adjacent controls	Actinobacteria ↓	[22]

BC: Bladder cancer; HC: healthy controls; UC: urothelium carcinoma.

Up to now, there are some studies concentrating on the possible relevance between urinary microbiota and bladder cancer. Urinary microbiota in bladder cancer patients was demonstrated to be distinguished from that of healthy controls, characterized by a decreased diversity, evenness, and richness^[14]^. Xu *et al*. pointed out that the abundance of *Streptococcus* increased in urine from cancer patients through a small-sample study involving 8 urothelial cancer patients and 6 healthy controls^[15]^. Bučević Popović *et al*. found that the urinary microbiota of both 12 male patients with bladder cancer and 11 healthy controls showed enrichment in *Firmicutes*, *Actinobacteria*, *Bacteroidetes*, and *Proteobacteria*^[16]^. Meanwhile, there was no difference in microbial diversity and overall composition between the two groups, but the abundance of *Fusobacterium*, *Actinobaculum*, *Facklamia*, and *Campylobacter* in the urinary microbiota of the cancer group was significantly elevated. Wu *et al*. demonstrated the abundance of *Acinetobacter*, *Anaerococcus*, *Rubrobacter*, *Sphingobacterium*, *Atopostipes*, and *Geobacillus* presented a higher level in urine from bladder cancer patients, along with significantly increased microbiota richness in the cancer group^[17]^. These publications are inconsistent, which may be attributed to clinical demographic characteristics. For instance, sex is a key factor and it was reported that urinary microbiota showed different enrichment species between male and female patients with bladder cancer^[18]^. In addition, insufficient quantity of samples, varying urine sample collection methods, and contamination could account for the discrepancy. The heterogeneity of the urinary microbiota in different healthy individuals^[6]^ indicates that identifying bladder cancer-associated microbiota solely through a statistical approach is difficult, so pinpointing the role of certain microbes in tumorigenesis is still a great challenge.

The ability of urine microbiota to reflect tumor tissue microbiota is an ongoing controversial topic^[18,19]^. Therefore, it is essential to examine intratumoral microbiota in bladder cancer due to emerging evidence that suggests its metabolic activity and functional significance^[20]^. Pederzoli *et al*. detected an enrichment of *Burkholderia* in bladder cancer tissue compared to non-neoplastic tissue^[18]^. Mansour *et al*. showed that the microbiota in the mucosal tissue of bladder cancer patients was different from that in urine samples, and the abundance order from high to low was *Firmicutes*, *Actinobacteria*, *Proteobacteria*, *Bacteroidetes*, and *Cyanobacteria*, respectively^[19]^. Among them, *Akkermansia*, *Bacteroides*, *Clostridium sensu stricto*, *Enterobacter*, and *Klebsiella*, as “five suspect genera”, were significantly increased in bladder cancer tissue compared to urine samples, indicating their potential involvement in the onset of bladder cancer. Liu *et al*. found significantly decreased *Firmicutes* and *Bacteroidetes*, together with increased *Proteobacteria* and *Actinobacteria* at the phylum level in cancer tissue^[21]^. Concurrently, *Cupriavidus spp*, *unclassified Brucellaceae*, *Acinetobacter*, *Escherichia‐Shigella*, *Sphingomonas*, *etc*. were significantly increased at genus level in tumor tissue compared to noncancerous tissue. Although there was no statistically significant difference in α-diversity between the two groups, the lower degree of bacterial diversity in tumor tissue compared to noncancerous controls was apparent based on differences observed in β-diversity. Parra-Grande *et al*. found that the *Actinobacteria* showed stark differences when comparing tumor tissue and adjacent normal tissue and pointed out that the diversity of the microbiota in bladder cancer mucosal tissue was lower than that of paired normal controls^[22]^. Theoretically, focusing on the comparison between intratumoral microbiota and paired tissue microbiota seems to pave the way for further causal research and exploration of potential mechanisms.

Current studies of urinary tract microbiota are mostly based on 16S rRNA variable region sequencing, which can detect a small number of bacteria but has limitations, such as the inability to distinguish between live and dead bacteria or to detect nonbacterial organisms, including fungi and viruses^[23]^. Sequencing based on variable regions of 16S rRNA is sufficient to identify genera, but they are unlikely to distinguish species precisely^[24]^. Therefore, most of the current bladder cancer microbiome characterization reflects the genus level of microbiota composition, which may not represent the true species richness of microbiota samples. No statistically significant association was found between urinary tract infection and bladder cancer in the past^[25]^, which may need to be confirmed by further high-quality studies concentrating on specific bacterial species’ biological function in the urothelial local environment. Therefore, mapping specific microbes may be necessary to establish a precise link between urinary microbiota dysbiosis and tumors, similar to gut microbiota and colorectal cancer.

### Possible mechanisms that microbiota promotes tumorigenesis of bladder cancer

In the past, researchers rarely identified directly carcinogenic microbes, such as *Helicobacter pylori*, but increasing evidence indicates a type of “complicit microbes” that promote tumorigenesis without directly causing cancer^[20]^. This reminds us that microbe-meditated tumorigenesis may be related to dysbiosis of the microbiota and an inappropriate host response, rather than simply be attributed to a single specific pathologic microbe^[26,27]^. The microbiota in the urinary tract provides us with new insights about the carcinogenesis of the bladder; whether some “special” microbes in the urinary tract are highly related to the occurrence of bladder cancer in carcinogenic or protective mechanisms requires further study.

The mechanisms of how microbiota promotes tumorigenesis can be concluded as epithelium barrier destruction, inflammation, induction of gene mutations, influence of intracellular signaling transduction pathways for pro- or antitumor effects, *etc*., based on advances in different cancers^[12,26]^.

**Figure 1:**
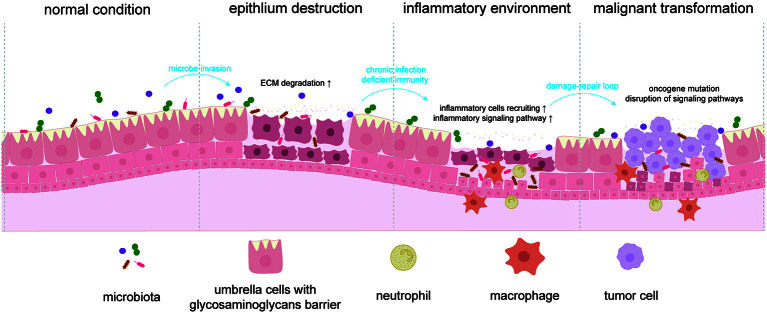
Possible mechanisms that microbiota promotes initiation of bladder cancer. Lesion starts with invasion of microbiota, which usually damages epithelial barrier through multiple virulence factors^[31,40]^ and increases the chance that microbiota interacts with bladder cells. Formation of intracellular bacteria and incomplete clearance^[41]^ from innate immune cells leads to chronic infection with an inflammatory environment characterized by various inflammatory mediators and immune cells. Chronic barrier destruction leads to more interactions between microbes and host, and persistent damage-repair loop (an inappropriate host response) of bladder epithelium plus inflammation environment increases the possibility of gene mutation and disrupts the intracellular signaling pathways^[36]^. Overall, multiple microbiota-mediated hits may contribute to the carcinogenesis of bladder cancer and further research needs to evaluate this relevance.

Extracellular matrix (ECM) is a key component supporting cells to behave well biologically, and it is considered to play a crucial role in carcinogenesis, progression, and metastasis in cancer^[28,29]^. Normally, in the bladder, the umbrella cell layer covered by a glycosaminoglycan^[30]^ (also the component of ECM) barrier touches the urine and commensals directly, which means the epithelium is separated from microbes and hemostasis is maintained if there are no invasive pathogens. Some bacteria produce virulence factors, including collagenases, hyaluronidases, elastase, *etc*., to cause ECM degradation^[31]^, including the glycosaminoglycan barrier and conjunction of cells, promoting bacteria spread and eventually leading to epithelium destruction and exfoliation. This process may cause severe inflammation response and obvious symptoms such as hematuria and lower urinary tract symptoms, thus prompting people to medical antibiotics treatment (the concept “urinary tract infection”). However, when the immune system fails to completely clear the infection, for instance, due to the formation of intracellular quiescent bacteria communities^[32]^, the unresolved and chronic inflammation attributed to a bacteria infection is characterized by induction of inflammatory pathways, various inflammatory mediators (cytokines, chemokines) and recruiting of immune cells, and this chronic stimulation is identified as a risk factor of bladder cancer^[33,34]^. Observations of reduced expression of Toll-like receptors (TLRs) in bladder cancer imply a possible role of microbiota in this immune-deficient situation^[35]^. Persistent damage from microbiota, disruption induced by inflammation, and continuous regeneration of bladder epithelial cells cause genomic instability and increase the possibility of gene mutation^[36]^. Additionally, the inflammatory environment disrupts the intracellular signaling pathway, especially STAT3 signaling, which is demonstrated to play a crucial role in the occurrence and proliferation of bladder cancer^[37-39]^. Multiple hits and steps induced by microbiota may contribute to the initiation of bladder cancer [Figure 1].

Currently, a pathogen with a clear carcinogenic effect in the bladder is *Schistosoma*, which is not a microbe but a parasite. Chronic bladder schistosomiasis induces local inflammation, fibrosis, and granulomas histologically. And the chemical products of *Schistosoma* (such as catechol-estrogen-like metabolites) can damage DNA directly, or indirectly through oxidative stress, leading to the accumulation of mutations in genes including *RAS*, *TP53* and others, and eventually inducing a malignant transformation of bladder epithelial cells^[42]^. *Bacteroides* and *Enterococcus* were highly abundant in bladder cancer tissue^[19,22]^. Previous studies demonstrated that enterotoxigenic *Bacteroides fragilis* in the gut cleaves E-cadherin on the surface of colonic epithelial cells by *B. fragilis* toxin, which causes proliferation and cancerous transformation of colonic epithelial cells through the β-catenin signaling pathway^[43]^. In addition, enterotoxigenic *Bacteroides fragilis* triggers IL-17R, NF-κB, and STAT3 inflammatory pathways that play key roles in colon carcinogenesis^[44]^. Similarly, *Enterococcus faecalis* in the gut induces M1 polarization of macrophages and activates the Wnt/β-catenin pathway, leading to reprogramming and dedifferentiating colonic epithelial cells and driving the initiation of colorectal cancer^[45]^. The Wnt/β-catenin signaling pathway plays an important role in the initiation, invasion, metastasis, and epithelial-mesenchymal transition of bladder cancer, confirmed by numerous researches^[46-48]^. *In vitro* and animal experiments have also found that silencing STAT3 has an inhibitory effect on bladder cancer^[49]^. So, whether chronic bladder transitional epithelium infection by these bacteria leads to malignant transformation of epithelial cells through a similar cancer-promoting molecular mechanism deserves further study.

## MICROBIOTA AND PROGRESSION OF BLADDER CANCER

### The composition of microbiota in different tumor staging

Tumor grading and staging are the basis of the clinical treatment of cancer. Tumor grading, a histology concept, depends on histopathologic slides, while the staging is more of a clinical application and is relevant to the outcome of cancer.

Bladder cancer can be classified as non-muscle invasive bladder cancer (NMIBC) and muscle invasive bladder cancer (MIBC), and it is reported that no striking difference was validated when comparing the composition of urinary microbiota between NMIBC and MIBC^[19]^. Contradictorily, Chipollini *et al*. detected significant enrichment of *Bacteroides* and *Faecalibacterium* in MIBC group compared to NMIBC group^[14]^. Wu *et al*. found that different risk levels stratified by EORTC (European Organization for Research and Treatment of Cancer) were related to different spectrums of urinary microbiota^[17]^. An overrepresentation of *Herbaspirillum*, *Porphyrobacter*, and *Bacteroides* was detected in bladder cancer patients with a high risk of recurrence and progression, suggesting that these genera could be utilized for risk stratification and prediction of cancer prognosis. Oresta *et al*. discovered that with the progression of bladder cancer, the abundance of *Veillonella* and *Corynebacterium* in the urine increased significantly^[50]^. Parra-Grande *et al*. pointed out that the abundance of *Enterococcus* in low-grade bladder cancer tissue was significantly higher than that in high-grade cancer tissue^[22]^.

The inconsistent evidence [Table 2] did not suggest potential microbial biomarkers associated with bladder cancer progression. As mentioned before, 16S rRNA sequencing of the urinary microbiota is not precise enough to identify species, which may ignore changes in the abundance of specific bacteria in the urine. The intratumoral and urinary microbiota are not completely equivalent, but urinary microbial signatures associated with bladder cancer progression appear to have more clinical potential because urine, rather than bladder cancer tissue, is an easily accessible specimen clinically.

**Table 2:** Urinary or intratumoral microbiota in different tumor staging of bladder cancer

**Research**	**Sample**	**Microbiota related to staging**	**Ref.**
Chipollini *et al*. 2018	Urine samples from 38 UC and 10 HC	MIBC group: Bacteroides ↑ Faecalibacterium ↑	[14]
Wu *et al*. 2018	Urine samples from 29 BC and 18 HC	Low-grade tumor: Enterococcus ↑	[17]
Mansour *et al*. 2020	10 BC urine samples and 14 BC tissue samples	No difference between NMIBC and MIBC	[19]
Parra-Grande *et al*. 2022	32 BC tissue samples and 26 adjacent controls	High risk of progression group: Herbaspirillum ↑ Porphyrobacter ↑ Bacteroides ↑ Marmoricola ↑	[22]
Oresta *et al*. 2021	/	With the progression of BC: Veillonella ↑ Corynebacterium ↑	[50]

BC: Bladder cancer; HC: healthy controls; MIBC: muscle invasive bladder cancer; NMIBC: non-muscle invasive bladder cancer; UC: urothelium carcinoma.

### Interactions between microbiota and tumor microenvironment

**Figure 2:**
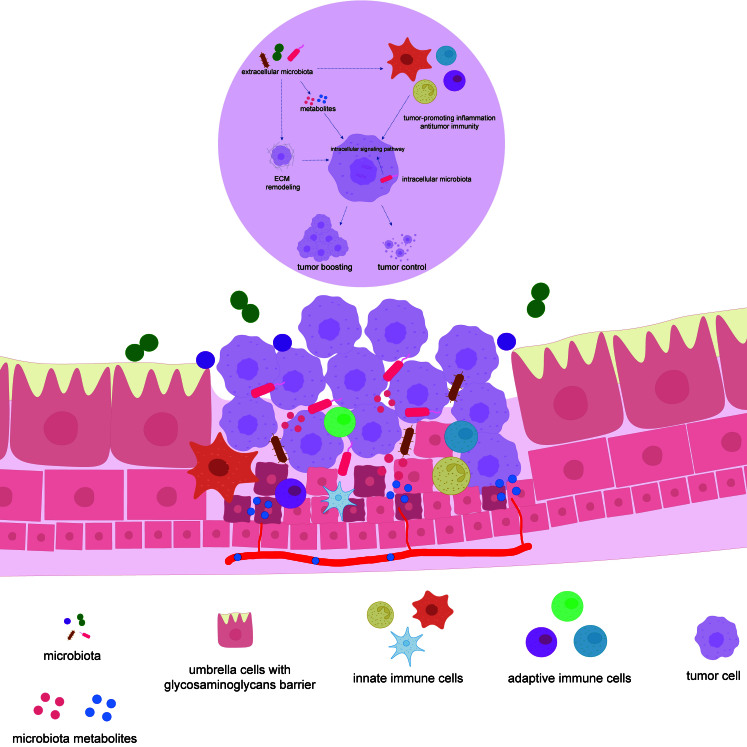
Possible overview of microbiota in the TME of bladder cancer. Extracellular microbiota may directly influence tumor cells through metabolites^[53]^ or ECM remodeling^[54]^. Gut microbiota is able to produce a remote effect through systemic microbial metabolites^[55-58]^. Furthermore, the microbiota can interplay with the immune compartment in the TME and result in immunomodulation^[59]^. Notably, the intracellular microbiota may influence the gene expression or intracellular signal pathways to manipulate the behavior of tumor cells^[52]^. The blue metabolites refer to the systemic microbiota metabolites. ECM: Extracellular matrix; TME: tumor microenvironment.

The crosstalk among different components of a tumor is intricate. Emerging evidence suggests microbiota is an unexpected participant in the tumor microenvironment (TME), even in some types of tumors that appear to be “germ-free”. In 7 kinds of solid tumors, such as breast cancer and brain tumor, microbes were detected inside tumor cells and immune cells and presented to be in a cell wall-deficient state similar to L-form bacteria^[51]^. Intratumoral microbial communities are located in microniches that are less vascularized and more immunosuppressive^[52]^, suggesting that the distribution of microbes in tumor tissue is not random but rather prefers a compatible niche in ECM or inside cells. In a word, intratumoral microbes located in different microniches may have various active states and functional responses. Considering the evidence that there exists microbiota inside bladder cancer tissue, we hope to recapitulate the possible function of microbiota in the TME and put forward potential mechanisms [Figure 2].

Commensals living in bladder mucosa may interact with the tumor immune microenvironment and promote bladder cancer progression. Innate immunity, including pathogen-associated molecular pattern (PAMP) and innate immune cells, probably contributes to bladder cancer development. Uropathogenic *Escherichia coli* (UPEC) is the most common microbe in urinary tract infection^[40]^, and UPEC infection upregulates the expression of cytokines such as IL-6 and IL-8 in urothelial cells through the TLR-4 pathway^[60]^. IL-6 induces antimicrobial peptide expression to promote the clearance of UPEC, and IL-8 promotes neutrophil chemotaxis towards the site of infection to engulf UPEC^[61]^. Of note, IL-6 promotes the survival and proliferation of tumor cells and attenuates the antitumor ability of tumor-infiltrating immune cells through the IL-6/JAK/STAT3 signaling pathway in the TME^[62]^. Elevated expression of STAT3 is also linked to proliferation and invasion of bladder cancer^[63]^. Similarly, IL-8 can enhance tumor proliferation through the CXCR1/2 receptor on the tumor cell, thus recruiting myeloid-derived suppressor cells (MDSC) and neutrophils to TME, where an immunosuppressive microenvironment is formed^[64]^. Furthermore, microbiota may induce commensal-specific memory T cells that cross-react with tumor-associated antigens^[65]^, thus influencing the outcome of cancer. So, this forms a possible potential mechanism of the microbiota-immune-tumor axis in the TME parallel with opposing responses of tumor-promoting inflammation and antitumor immunity induced by tumor cells. Analogous mechanisms through the microbiota-immune-tumor axis have been revealed in other cancers, for instance, the microbiota is able to promote cancer development via γδT cells in lung cancer^[66]^. Hence, whether the microbiota-mediated inflammatory or immune response plays a pivotal role in the progression of bladder cancer remains unclear and further investigations should evaluate the validity of this hypothesis.

Microbial metabolites, especially those produced by gut microbiota, have been extensively investigated and provided insights into the relationship between gut microbiota and the progression of extraintestinal cancers. The gut microbiota-produced microbial metabolites, including tryptophan-derived microbial metabolites, bile acid, trimethylamine N-oxide (TMAO), short-chain fatty acids (SCFAs), *etc*. are identified to regulate various components in the TME. Many compounds produced by tryptophan metabolism play a significant role in inflammation and immune response^[67]^. Indole-3-acetic acid, a tryptophan metabolite produced by gut microbiota, was reported to activate tumor-associated macrophage to suppress the CD8+ T cell-mediated antitumor immunity in a pancreatic tumor model^[55]^, while another one, indole-3-aldehyde, can activate CD8+ T cells to proliferate and release IFN-γ to enhance antitumor immunity in a melanoma model^[56]^. *Lactobacillus reuteri* administration suppresses colorectal tumorigenesis via the tryptophan catabolite-indole-3-lactic acid (ILA), which exerts antitumorigenic effects by downregulating the IL-17 signaling pathway^[68]^. In addition, indoleamine 2,3-dioxygenase 1, a key enzyme of tryptophan metabolism, was demonstrated to promote antitumor immunity and inhibit angiogenesis in bladder cancer^[69]^. A study revealed that plasma tryptophan level was significantly decreased in bladder cancer patients^[70]^. So, whether systemic tryptophan-derived microbial metabolites have an impact on TME of bladder cancer needs to be further explored. Bile acids that are metabolized by gut microbiota and the liver (this forms enterohepatic circulation of bile acid) play a pivotal role in maintaining healthy gut microbiota and innate immunity, and are linked with carcinogenesis of colorectal cancer and hepatocellular carcinoma^[57,58]^. However, bile acids, including chenodeoxycholic acid (CDCA), Glycoursodeoxycholic acid (GUDCA), and glycochenodeoxycholic acid (GCDCA), were detected to be upregulated in urine samples of bladder cancer patients compared to healthy controls^[71]^. Furthermore, farnesoid X receptor (a nuclear receptor that can be activated by binding with bile acids) was reported to inhibit the migration, invasion, and angiogenesis of bladder cancer *in vitro* through various mechanisms^[72,73]^. This reminds us that the bile acids in circulation or urine may partly influence the progression of bladder cancer. TMAO is broadly studied in cardiovascular diseases such as atherosclerosis and heart failure. However, recent publications revealed that the microbiota-derived metabolite TMAO also activates CD8+ T cells and promotes antitumor immunity in breast cancer^[74]^ and pancreatic cancer^[75]^. In addition, microbial SCFAs enhance the antitumor activity of cytotoxic T lymphocytes (CTLs) and chimeric antigen receptor (CAR) T cells in syngeneic murine melanoma and pancreatic cancer models by increasing the production of effector molecules such as CD25, IFN-γ and TNF-α^[76]^, indicating complicated crosstalk between microbial metabolites and cytokines. Until now, no evidence showed a connection between microbial metabolites, including TMAO and SCFAs, and the progression of bladder cancer. Although the progression of bladder cancer has not been demonstrated to be linked with systemic change of microbial metabolites in circulation or accumulation of microbial metabolites in urine, more and more evidence implies that gut microbiota may exert a remote effect to influence the TME of bladder cancer.

ECM remodeling in the TME could be regulated by tumor cells, cancer-associated fibroblasts (CAFs), inflammatory cells, *etc*. and was highlighted to a significance of cancer development, especially invasion and metastasis. As discussed before, some microbiota can produce virulence factors that cause ECM degradation, suggesting a potential mechanism that intratumoral microbiota influence the tumor cells in an ECM remodeling-dependent manner. Matrix metalloproteinases (MMPs), the proteolytic enzymes inducing ECM degradation and remodeling, are vital for the progression of bladder cancer, and influence tumor cell proliferation, apoptosis, invasion, and metastasis^[77]^. *Eubacterium sp.* cocultured with bladder cancer organoids, which has been proved to retain the histological and genomic features of parental tumors^[78]^, can promote tumor cell proliferation through the ECM1/ERK1/2 phosphorylation/MMP9 pathway, and this microbe is found related to bladder cancer in NMIBC cohort clinically^[54]^.

Intracellular microbiota in the TME has an impact on the progression of bladder cancer in unique ways. Prior research in other cancers revealed complex functions of the intracellular microbiota. Intracellular bacteria can affect cancer cell invasion, metastasis, and repair of DNA damage based on a study of oral squamous cell carcinoma and colorectal cancer cells^[52]^. Some types of cancers, such as melanoma cells^[59]^, can present intracellular bacterial peptides through HLA molecules, shaping the tumor immune microenvironment and influencing T cell reactivity. Intracellular bacteria are emphasized to infect the urothelial layer and the tumor cells, and this could be a prominent target for humoral immunity, linking immune memory cells to response to non-microbial tumor antigens^[79]^, but the mechanisms are poorly understood. The formation of intracellular bacterial communities in chronic infectious urothelial epithelial cells implies that the intracellular microbiota in bladder cancer might disturb the intracellular signaling pathways, or relevant microbial antigens could be present at the surface of the cell membrane of bladder cancer and interact with the immune compartment of TME, although there is no evidence about the existence and functional activity of intracellular bacteria of bladder cancer so far.

## MICROBIOTA AND TREATMENT OF BLADDER CANCER

The discovery of intratumoral microbiota is the milestone of a comprehensive understanding of the tumor microenvironment, and it has been proved that microbiota plays a potential role in mediating tumor resistance to drugs^[80]^.

### Relationship between microbiota and recurrence of bladder cancer

The standard treatment option for patients with NMIBC is transurethral resection, and whether the subsequent intravesical instillation treatment is necessary depends on the assessment of pathohistological slides and other risk factors, with 5-year recurrence rate ranging from 20% to more than 50% according to different risk groups to which the patients belong^[81]^. The recurrence mechanisms of urothelial cancer can be recapitulated into five points, including undetected cancer at cystoscopy, local residual disease after TURBT, tumor reimplantation, drop metastasis from upper tract urothelial carcinoma, and field change cancerization effect^[82]^. As a urine storage organ, the bladder is exposed to the urine with the accumulation of carcinogens such as cigarette metabolites and chemicals, which induces multiple independent lesions with precancerous genetic changes, leading to multiple tumors or recurrence. This prompts us to consider whether the potential presence of microbes in urine or bladder mucosa also has this field-change effect. To investigate this, we have compiled and summarized observational studies to explore whether part of the microbiota is linked to the recurrence of bladder cancer [Table 3].

**Table 3:** Urinary or intratumoral microbiota relevant to recurrent bladder cancer and response to BCG

**Research**	**Sample**	**Microbiota related to recurrence**	**Microbiota related to BCG response**	**Ref.**
Wu *et al*. 2018	Urine samples from 29 BC and 18 HC	Herbaspirillum ↑ Gemella ↑ Bacteroides ↑ Porphyrobacter ↑ Faecalibacterium ↑ Aeromonas ↑		[17]
Zeng *et al*. 2020	Urine samples from 40 NMIBC	Anoxybacillus ↑ Massilia ↑ Thermomonas ↑ Brachybacterium ↑ Micrococcus ↑ Nocardioides ↑		[86]
James *et al*. 2023	Urine samples from 29 NMIBC	Aerococcus ↑ Ureaplasma ↓ Escherichia/Shigella ↓		[87]
Knorr *et al*. 2021	Tissue samples from 26 BC		Corynebacterium ↑ Pseudomonas ↑	[91]
Knorr *et al*. 2022	Tissue samples from 47 BC		Lactobacillus ↑ Corynebacterium ↓	[90]
Hussein *et al*. 2021	Urine samples from 43 BC and 10 HC		Serraia ↑ Brochothrix ↑ Negativicoccus ↑ Escherichia-Shigella ↑ Pseudomonas ↑	[92]

BC: Bladder cancer; BCG: Bacillus Calmette-Guérin; HC: healthy controls; NMIBC: non-muscle invasive bladder cancer; UC: urothelium carcinoma.

In the early 1990s, a clinical study found that oral administration of *Lactobacillus casei* preparation had a protective effect on the recurrence of bladder cancer after surgery^[83,84]^. Although the mechanism of *Lactobacillus casei* intervention in the recurrence of bladder cancer was unclarified then, this study showed the prospect of microbial application in the treatment of bladder cancer. Patients with asymptomatic bacteriuria (defined as the presence of bacterial growth on clinical standard urine culture but no symptoms of infection) in low-grade papillary NMIBC have a significantly lower recurrence rate than those without infection^[85]^, which may be related to innate immunity activated by bacteria and suggests the potential of the urinary microbiota for predicting tumor recurrence in clinical practice. However, this observational study did not identify the composition of the urinary microbiota. Further investigation of urinary microbial species showed that patients with NMIBC had an elevated risk of recurrence with a higher bacterial richness in urine samples; *Herbaspirillum*, *Gemella*, *Bacteroides*, *Porphyrobacter*, *Faecalibacterium*, and *Aeromonas* may be potential predictive biomarkers^[17]^. Inconsistently, Zeng *et al*. found that the abundance of 9 species of bacteria, including *Anoxybacillus*, *Massilia*, *Thermomonas*, *Brachybacterium*, *Micrococcus*, and *Nocardioides*, was significantly increased in the NMIBC patients with recurrence^[86]^. A study concentrating on Bacillus Calmette-Guérin (BCG)-treated NMIBC patients noted a higher relative abundance of *Aerococcus* in urine from patients with relapse, while *Ureaplasma* and *Escherichia/Shigella* elevated in urine from patients without relapse^[87]^. A healthy gut microbiota is often characterized by high richness^[88]^, but a high richness of microbial communities in the urinary system may not be a healthy phenomenon. The significantly increased richness of the urinary microbiome suggests a higher risk of recurrence, but whether specific responsible bacteria can be identified to quantitatively assess this risk still needs further research and clinical verification.

### Microbiota and BCG intravesical instillation

BCG is a type of vaccine made up of an attenuated strain of *Mycobacterium bovis*. BCG intravesical instillation is the gold-standard adjuvant treatment for NMIBC with a high risk of progression and induces initial complete response rates of 55%-65% for high-risk tumors and 70%-75% for carcinoma *in situ*^[89]^. Although *Mycobacterium bovis* is not normally present in the urinary tract, its unique role described in the treatment of bladder cancer highlights the relationship between microbes and antitumor immunity, more precisely, the relationship between microbiota and tumor immune microenvironment.

The link between urinary microbiota and BCG response remains controversial [Table 3]. Knorr *et al*. demonstrated a significantly increased abundance of *Lactobacillus* in BCG responders and *Corynebacterium* in BCG non-responders^[90]^, which contradicts their previous conclusion of the enrichment of *Corynebacterium* in BCG responders^[91]^. Hussein *et al*. reported that the abundance of *Serraia*, *Brochothrix*, *Negativicoccus*, *Escherichia-Shigella*, and *Pseudomonas* was significantly increased among NMIBC patients who responded to BCG treatment^[92]^. The above evidence did not identify consistent bladder microbes that may be associated with BCG response, but suggested that the bladder microbiota may influence the antitumor effect of BCG. Current comprehension of underlying mechanisms of BCG in treating bladder cancer includes direct cytotoxic effects and complicated antitumor immune responses that rely on attachment and invasion of *Mycobacterium bovis* successively^[89,93]^. The commensal microbes in the bladder may impact the attachment and invasion of *Mycobacterium bovis* through colonization, metabolites or interact with the immune cells, resulting in a synergistic or antagonistic effect on antitumor immunity.

### Microbiota and immune checkpoint inhibition therapy

The immune checkpoint inhibition therapy (CPI) blocks immunosuppression induced by tumor cells through competitively binding to receptors such as programmed cell death protein 1 (PD-1) and cytotoxic T lymphocyte-associated protein 4 (CTLA-4) on immune cells, allowing tumor-infiltrating immune cells to exert antitumor effects^[94]^. The objective response rate (ORR) of immune checkpoint inhibitor monotherapy in patients with metastatic bladder cancer who are intolerant to platinum-based chemotherapy is only 20%-30%^[95-97]^.

Gut microbiota has been demonstrated to be associated with the efficacy of CPI in different cancers. For instance, the gut microbiota is required for the antitumor effect of CTLA-4 blockade in melanoma and colon cancer^[98]^. *Bifidobacterium pseudolongum*, *Lactobacillus johnsonii*, and *Olsenella* species living in the gut are proved to significantly enhance the efficacy of immune checkpoint inhibitors, including CTLA-4 and PD-1 inhibitors in bladder cancer, melanoma, and intestinal cancer murine models, which is dependent on metabolite inosine^[99]^. This prompts consideration of whether the urinary microbiota has a similar impact on bladder cancer, but considering the gut microbiota constitutes the largest microbial reservoir in the human body, it appears more plausible that local metabolic activity induced by urinary microbiota would occur rather than systemic alteration induced by it. A clinical study found that the administration of antibiotics attenuated the response to Pembrolizumab in NMIBC patients and was related to a lower rate of complete response and recurrence-free survival^[100]^, suggesting a prominent clinical impact of microbiota on immunotherapy of bladder cancer. However, the role of urinary microbiota in the immunotherapy of bladder cancer remains uncertain due to the potential perturbation of both urinary and gut microbiota by various patterns of antibiotic metabolism. Furthermore, the gut microbiota has been shown to affect both intestinal and extraintestinal cancers^[98,99,101,102]^, which makes it more complicated to identify whether gut microbiota or urinary microbiota eliminated by antibiotics influences the efficacy of immunotherapy. Characterization of the urinary microbiome has identified some bacteria that may be associated with CPIs in bladder cancer. Chen *et al*. observed that *Leptotrichia*, *Roseomonas*, and *Propionibacterium* were enriched in the urine of PD-L1-positive NMIBC patients, while *Prevotella* was enriched in PD-L1-negative patients, suggesting that these bacteria genera may affect the responses of CPI^[103]^. However, in fact, PD-L1 expression in tumor or immune cells assessed by immunohistochemistry does not appear to be consistent with the response to checkpoint inhibitors, and its use as a predictive marker is controversial^[104]^. The association between microbiota and antitumor immunity in bladder cancer requires additional investigation in preclinical research.

Patients with metastatic bladder cancer have lost the chance to undergo surgery, leading to the selection of systematic chemotherapy or immunotherapy as viable treatment options. Currently, treatment selection cannot rely on predictive biomarkers as these biomarkers have not consistently differentiated patient groups for treatment and are thus discouraged for clinical application^[81]^.

### Microbiota-based clinical interventions in bladder cancer

Microbiota represent complicated effects in cancer therapy. Removing carcinogenic bacteria like *Helicobacter pylori* in the stomach has great significance in preventing gastric cancer, while the use of antibiotics attenuates the therapeutic effect of CPI in melanoma. Up to now, microbiota is more of therapeutic importance in bladder cancer. BCG intravesical instillation is broadly used as postoperative follow-up treatment of bladder cancer, though it has many local and systemic side effects, such as bacterial or chemical cystitis, contracted bladder, fever, and general malaise^[105]^. Hence, it is necessary and promising to develop novel antitumor drugs that are instilled into the bladder and probiotics seem a reasonable choice. Intravesical instillation of Ty21a (a vaccine against typhoid fever) was demonstrated to control bladder cancer through a dendritic cell and T cell-dependent manner in mice model and a relevant phase I trial is underway(NCT 03421236: IVES Ty21a)^[106]^. Systemic metabolites produced by gut microbiota are capable of modulating treatment efficacy in multiple kinds of cancers, based on preclinical experiments where oral gavage with microbiota or precursors of microbial metabolites controls tumor growth, suggesting oral probiotics or relevant microbial metabolites are promising in cancer therapy^[55,56,74]^, but this hypothesis needs verification through clinical trials. There is growing evidence indicating that the microbiota has a synergistic effect^[98,99,101,102]^ with immunotherapy on immunomodulation of the TME, and this might be applied to anti-PD-1 therapy in metastatic bladder cancer, addressing its limited efficacy in advanced patients’ outcomes.

In conclusion, both intratumoral microbiota and gut microbiota could be a potential therapeutic target for cancer. Special treatment strategies to manipulate microbiota composition in bladder cancer (such as intravesical instillation or drugs metabolized through the kidney) deserve attention.

## CONCLUSION

After the discovery that urine is no longer sterile, numerous studies aimed to characterize the microbiota have observed high variability in urine and bladder cancer tissue between different individuals, which brings greater challenges to the investigation of microbes in pathological conditions. Furthermore, the concept of “asymptomatic bacteriuria” seems to be partly explained due to the progressive understanding of urinary microbiota. Different studies suggest some genera, such as *Anaerococcus*, under the phylum *Firmicutes* and *Acinetobacter*, may be responsible for the development of bladder cancer, but further preclinical studies are needed to verify. The bacteria that are not detected precisely enough to distinguish spices may be attributed to the insufficient accuracy of 16S rRNA sequencing technology, which makes it challenging to elucidate the potential role of specific microbiota in bladder cancer. Urinary microbes may serve as potential biological predictors to assess the chances of bladder cancer recurrence and the effectiveness of treatment, but confirmation of their clinical application value requires further study.

Microbiota has been proved to be related to tumorigenesis and progression in different types of tumors. Currently, only chronic cystitis caused by *Schistosoma* infection has a causality relationship with bladder squamous cell carcinoma, suggesting that chronic inflammation plays an important part in the malignant transformation of urothelial cells. There are few studies directly revealing how a certain type of microbe influences the biological behaviors of bladder cancer, hindering our comprehension of the role of microbiota in this disease. The tumor microenvironment is closely related to tumor cell proliferation, invasion, metastasis, and other biological behaviors^[107]^. Increasing pieces of evidence elucidate that microbiota is also identified to be one of the key components of the tumor microenvironment, and it can exist in different microniches of the tumor microenvironment and perform different functions. The balance between pro-tumor inflammation and antitumor immunity plays an important role in tumor progression^[108]^, and some special microbes may interact with the host through innate immune response to skew this balance. Therefore, the immune or inflammatory response acts like a bridge in the study of the effect of microbiota on bladder cancer. The application of BCG in the treatment of bladder cancer represents an example of microbe-mediated antitumor immunity, although the mechanism has not been fully elucidated. The gut microbiota can extensively influence the efficacy of immune checkpoint inhibitors in intestinal and extraintestinal cancer, suggesting a systemic change induced by gut microbiota metabolism. The intratumoral microbiota has been identified to influence multiple components of the tumor microenvironment, including tumor cells and immune cells^[45,66,109,110]^, thus leading to boosting or control of tumor development. Are both the bladder and gut microbiota involved in local immune regulation in bladder cancer? Does bladder cancer-associated microbiota have therapeutic significance? Further research is needed to address these questions.

In summary, this review focuses on the current research on the microbiota in the urinary tract and describes the possible microbiota associated with the initiation, progression, and treatment of bladder cancer. Based on the advances in the relationship between gut microbiota and tumors including bladder cancer and other cancers, this review emphasizes the network formed by microbiota, microbial metabolites, immune (inflammatory) response, and tumor in bladder cancer tissue and discusses the possible mechanisms of how microbiota influences the biological behavior of bladder cancer. Finally, this review points out the potential clinical applications of bladder-resident microbiota and gut microbiota in treatment, including as an antitumor agent and affecting the efficacy of the immunotherapy.

## DECLARATIONS

### Copyright

© The Authors 2023.
